# Challenges of schistosomiasis control in Africa: The path ahead

**DOI:** 10.1002/puh2.166

**Published:** 2024-03-15

**Authors:** Ejovwokeoghene Joseph Omohwovo, Kebabonye P. Gabaake, Don Eliseo Lucero‐Prisno

**Affiliations:** ^1^ University of Port Harcourt Choba Nigeria; ^2^ Faculty of Health Sciences, School of Allied Health Professions University of Botswana Gaborone Botswana; ^3^ Department of Global Health and Development London School of Hygiene London UK; ^4^ Faculty of Management and Development Studies University of the Philippines Open University Los Banos Laguna Philippines; ^5^ Faculty of Public Health Mahidol University Bangkok Thailand; ^6^ Department of Global Health and Development London School of Hygiene & Tropical Medicine London UK

## Abstract

Schistosomiasis is a neglected tropical disease that is endemic in sub‐Saharan Africa, with over 90% of global cases occurring in the region. Poverty, inadequate healthcare infrastructure, and limited access to water and sanitation contribute to the high prevalence of the disease. Despite efforts to control and prevent the transmission of schistosomiasis, a reduction in the transmission rates has not been realized owing to several challenges. This article highlights the challenges of schistosomiasis control in Africa and recommends strategies for successful interventions. These strategies include prioritizing government backing, investing in academic partnerships, strengthening integrated health programs, targeting communities through the primary healthcare system, and adopting home‐grown solutions. Additionally, it is essential to address the knowledge gap hindering effective control of the disease and the risk of reinfection after treatment. By implementing these strategies, it is possible to achieve the World Health Organization's 2021–2030 goal of eliminating schistosomiasis as a public health problem and reducing its prevalence of heavy infection to less than 1%.

## INTRODUCTION

As of 2022, Africa's population stood at approximately 1.4 billion people, making it the second most populous continent after Asia [1]. Amidst this vast and diverse population, Africa grapples with a multitude of health challenges, including neglected tropical diseases (NTDs) that continue to pose significant threats to public health [2]. Among these diseases, schistosomiasis, also known as bilharzia, warrants attention due to its widespread prevalence and detrimental impact on communities across the continent [3]. Schistosomiasis is an NTD caused by parasitic flatworms. In Africa, two main species of *Schistosoma*, namely, *Schistosoma mansoni* and *Schistosoma haematobium*, are responsible for the majority of human infections [4]. *S. mansoni* is responsible for intestinal schistosomiasis, whereas *S. haematobium* causes urogenital schistosomiasis [4]. The prevalence of this disease in Africa is high, particularly in sub‐Saharan Africa (SSA). According to the World Health Organization (WHO), over 90% of schistosomiasis cases occur in this region [3]. The annual mortality for SSA due to *Schistosomiasis mansoni* is 150,000–280,000 [5]. High transmission areas record a prevalence rate of 60%–80% in school‐aged children (SAC) and 20%–40% in adults [6]. The high prevalence of this disease is due to factors, such as poverty, inadequate healthcare infrastructure, and limited access to essential resources [4].

In comparison to other diseases, NTDs, including schistosomiasis, are often overshadowed by the global attention given to HIV/AIDS, tuberculosis, and malaria [7]. However, its endemicity and chronic nature make it a persistent health issue that affects millions of people in Africa [4]. This disease has a profound impact on public health and socio‐economic factors [7]. Chronic infections can lead to anemia, organ damage (such as liver and bladder), impaired growth, and cognitive consequences [3]; coupled with the socio‐economic burden, they perpetuate the cycle of poverty and hinder the overall development of affected communities. The severe complications of schistosomiasis call for an exploration of the challenges faced in its control and eradication. Therefore, this article aims to delve into the challenges of schistosomiasis control in Africa and recommends several strategies for successful elimination.

## CHALLENGES OF SCHISTOSOMIASIS CONTROL IN AFRICA

To achieve the WHO's ambitious goal of eliminating NTDs, including schistosomiasis, in Africa by 2030 and reducing the prevalence of severe schistosomiasis infections to less than 1% [8], it is crucial to recognize the key challenges that the continent is facing in controlling the transmission of schistosomiasis.

Africa has a climate that is ideal for the reproduction and propagation of snails, making it a hotspot for schistosomiasis [9]. Although various factors contribute to the prevalence of schistosomiasis in Africa, the climatic conditions, characterized by high temperatures and abundant rainfall, play a significant role in creating favorable breeding conditions for snails [9]. The scarcity of praziquantel medication for treating schistosomiasis poses a significant obstacle [3]. In 2015, only nine African countries attained the treatment target (75%) of SAC [10]. Additionally, the risk of reinfection of the disease makes it challenging to limit the spread even after treatment [3].

Another significant challenge that Africa faces is poverty, which is exacerbated by poor economic policies [11]. Schistosomiasis is a disease closely linked to poverty and is both a cause and a consequence of poverty [12]. The disease is most common in areas with inadequate living conditions that promote poor access to clean water, sanitation, and hygiene [13]. Furthermore, individuals with poor socio‐economic conditions do not have access to quality healthcare [14], making them more vulnerable to contracting schistosomiasis.

The lack of adequate governmental support in Africa is a crucial issue that hinders the effective combat of infectious diseases, such as schistosomiasis. The absence of sufficient domestic funding for control programs and the inadequate mapping of the distribution of the disease and snails often lead to a decline in the effectiveness of control strategies over time. Moreover, the healthcare system in several African countries faces significant challenges, including limited access to sensitive and diagnostic methods, shortage of healthcare professionals, and insufficiently functioning health facilities, resulting in subpar healthcare service delivery [14]. The health system in Africa heavily relies on imported medicines to treat various infectious diseases [15], including schistosomiasis. Unfortunately, limited local manufacturing capacity results in high costs for imported drugs, rendering them inaccessible and unaffordable for the affected population, particularly vulnerable groups, including children, women, and the disabled. These factors contribute to the inadequate control of schistosomiasis, which leads to the high prevalence of schistosomiasis‐related morbidity and mortality on the continent.

In Africa, another major problem in controlling schistosomiasis is that most people lack sufficient knowledge and understanding about the risk factors for transmission and preventive measures. This knowledge gap hinders effective control of the disease. To compound the problem, the lack of continuous and comprehensive awareness campaigns by government bodies, NGOs, civil society, and other stakeholders leads to a gradual loss of effectiveness of the control strategies implemented.

## THE WAY FORWARD FOR CONTROL OF SCHISTOSOMIASIS IN AFRICA

The following measures are recommended to address the difficulties faced by African countries in controlling schistosomiasis transmission in the region:

First, African countries must prioritize government support for NTD interventions and control [14], including schistosomiasis. These measures are not only highly cost‐effective but also yield one of the highest returns on investment in public health [11]. However, the process of eliminating schistosomiasis is lengthy and demanding, which may lead to a decrease in support over time. Therefore, governments should ensure sufficient and consistent funding for schistosomiasis‐elimination programs while devising tactics to ensure judicious use of the funds. Moreover, new operational strategies must be developed to effectively control schistosomiasis in Africa. To achieve this, the power of community engagement should be harnessed, enabling a collaborative approach to address the specific needs of affected communities [16]. By actively involving these communities, healthcare providers can gain a deeper understanding of their requirements and preferences, allowing for tailor‐made interventions that align with the community's unique challenges.

Second, African countries must invest in academic partnerships and establish collaboration networks with countries in the developed world. This collaborative approach has the potential to bring about significant benefits for the region: facilitating knowledge exchange, technological advancements, and socio‐economic growth, thus enhancing the elimination of schistosomiasis in the continent. Therefore, scaling up cooperative interventions between African countries and other nations is imperative for reducing the burden of this disease in Africa. There is also a pressing need to intensify research efforts aimed at combating schistosomiasis. This includes the development of accurate, feasible, yet sensitive diagnostic methods and markers for the disease. Given the significant burden of schistosomiasis on affected populations and the risk of reinfection, research and development of vaccines against the disease should also be given utmost importance [17]. Additionally, effective vector control measures for snails in schistosomiasis‐endemic regions must be implemented to complement treatment strategies. In the context of a one‐health approach, recognizing the interconnectedness between the human, snail host, and the environment calls for a multipronged approach policy and guidelines for national control programs. Moreover, the complexity of schistosomiasis is embedded in space‐specific environmental systems, and the dynamic nature of these systems determines the prevalence and effectiveness of interventions [16].

Third, prioritizing efforts to strengthen integrated health programs in Africa is crucial as it addresses schistosomiasis and co‐existing health challenges. This necessitates investments in intelligence and surveillance systems, capacity building for healthcare workers, modern healthcare facilities, and revitalized health financing [14]. Targeted intervention strategies through the primary healthcare system and country‐specific solutions that consider the continent's diversity are also essential [11]. To ensure the widespread availability of praziquantel medication in Africa, efforts should be made to strengthen existing domestic pharmaceutical companies as well as establish new ones that meet international standards. Collaboration and assistance from the international community should also be extended to the African region, as this will improve the praziquantel drug supply throughout the continent, particularly in endemic areas.

Lastly, it is commendable that successful schistosomiasis control measures have been implemented in some African countries, including Egypt and Mauritius [3]. These successful endeavors have demonstrated the feasibility of scaling up treatment to the national level [3]. Therefore, other African countries must follow suit and implement comprehensive schistosomiasis control measures to prevent an increase in morbidity and mortality rates associated with the disease. There should also be continued efforts to ensure the development and implementation of rapid and sustained economic growth policies and programs to reduce poverty and ensure sustainable development in the region. Finally, equitable access to technology and innovations must be ensured to bridge gaps and enhance healthcare and overall development in the continent.

## CONCLUSION

The challenge of interruption and ultimate elimination of schistosomiasis transmission in Africa requires urgent attention. Poverty, inadequate healthcare infrastructure, and limited access to essential resources are formidable obstacles to effective control efforts. To eliminate schistosomiasis in the continent, African countries must prioritize collaborative partnerships, strengthen health systems, develop new diagnostic tools, and intensify national, cross‐border, and regional efforts. Moreover, allocating more funds and empowering and engaging communities will play pivotal roles in the fight against schistosomiasis. Thus, it is essential to strengthen and judiciously engage governmental health agencies, NGOs, communities, civil society, and other stakeholders in the fight against the disease in Africa.

## AUTHOR CONTRIBUTIONS

Ejovwokeoghene Joseph Omohwovo conceptualized the manuscript. Ejovwokeoghene Joseph Omohwovo and Kebabonye P. Gabaake wrote the original draft. Ejovwokeoghene Joseph Omohwovo, Kebabonye P. Gabaake, and Don Eliseo Lucero‐Prisno III reviwed and edited the manuscript.

## CONFLICT OF INTEREST STATEMENT

Don Eliseo III Lucero‐Prisno is the Editor‐in‐Chief of Public Health Challenges. Therefore, he was excluded from editorial decision‐making related to the acceptance of this article for publication in the journal.
